# Counting Is Easier while Experiencing a Congruent Motion

**DOI:** 10.1371/journal.pone.0064500

**Published:** 2013-05-15

**Authors:** Luisa Lugli, Giulia Baroni, Filomena Anelli, Anna M. Borghi, Roberto Nicoletti

**Affiliations:** 1 Department of Philosophy and Communication Studies, University of Bologna, Bologna, Italy; 2 Department of Communication and Economics, University of Modena and Reggio Emilia, Reggio Emilia, Italy; 3 Department of Education Sciences, University of Bologna, Bologna, Italy; 4 Department of Psychology, University of Bologna, Bologna, Italy; 5 Institute of Cognitive Sciences and Technologies, National Research Council, Roma, Italy; University of Tokyo, Japan

## Abstract

Several studies suggest that numerical and spatial representations are intrinsically linked. Recent findings demonstrate that also motor actions interact with number magnitude processing, showing a motor-to-semantic effect. The current study assesses whether calculation processes can be modulated by motions performed with the whole body. Participants were required to make additions or subtractions while performing (on-line condition) or after having experienced (off-line condition) an ascending or descending motion through a passive (i.e., taking the elevator) or an active (i.e., taking the stairs) mode. Results show a congruency effect between the type of calculation and the direction of the motion depending on: a) the off-line or on-line condition, b) the passive or active mode and c) the real or imagined task. Implications of the results for an embodied and grounded perspective view will be discussed.

## Introduction

We calculate during each phase of our lives: we try to understand what happens when we invest our money, when we try to keep time and when we have to go home taking the right bus at the right time. All these everyday activities involve numbers and the relation between numbers and their magnitude, which means counting. Different researches have consistently suggested that numerical magnitude is linked with the processing of spatial information [1], [2], to the extent that it has been postulated a common cortical metrics of time, space, and quantity [3]. A straightforward demonstration of the strong association between numbers and space can be found in the so-called SNARC (Spatial Numerical Association of Response Codes) effect [4]. In this task, participants are typically faced with numbers ranging from 1 to 9 and asked to make a parity judgment by pressing a left or right key. Results show better performances when responding with the left key to small numbers (e.g., 2) and with the right key to large numbers (e.g., 7), with respect to the opposite instructions. This evidence led Dehaene and co-authors to postulate the existence of an horizontal mental number line (MNL) where numbers are progressively located from left to right according to their magnitude (see [5], [6], [7], see also [8] for a different account). The existence of an horizontal MNL has gathered support by several evidence so far, while few studies have found a SNARC effect also for vertical number arrangements (i.e., a facilitation for upward or downward responses to large and small numbers, respectively) across different response modalities, such as key-presses [9] and eye movements [10]. Recently, Holmes and Lourenco [11] focused on the relative strength of the horizontal and vertical mental number organization and found that the vertical axis would be only triggered when numbers are conceptualized as magnitudes that elicit an orientation (e.g., 1^st^ floor from surface, 2^nd^ floor from surface, etc).

Despite the majority of the SNARC studies focused on the influence that number representation has on spatial attention [12], [13], [14], several researches have shown that numerical magnitude can also modulate action-related processes [15], [16], [17], [18], [19], [20]. Further studies demonstrated a bidirectional relation between numbers processing and action-related processes (i.e., motor-to-semantic effect, [21] ]23]). More specifically, the motor-to-semantic effect revealed a facilitation, in terms of response latencies, when participants observed a closing grip posture of a biological hand and had to generate small numbers. Badets et al. [21] claimed that the “motor-to-semantic effect is assumed to come from the action system of the participant who either actively performs an action or passively experience a motion and generates a number after” (*ibidem*, p. 2). Other studies have investigated the influence of the specific body parts' movements, such as head positions [24] or ocular saccades [25], on the generation of small numbers.

In our study we assess whether and to what extent motions experienced with the whole body can influence arithmetical calculations of addition and subtraction. We hypothesize, thus, that these calculations, both leading to numerical magnitudes, can be conceptualized along an upward and downward orientation for additions and subtractions, respectively. A study by Knops, Viarouge, and Dehaene [26] demonstrated that arithmetical calculations bias corresponding spatial location over others: participants tended to select the numerosity displayed in the upper right location for additions, and in the upper left location for subtractions (Space-Operation Association of Responses: SOAR). Differently from Knops and colleagues, we ask participants: (a) to keep adding or subtracting the same quantity (i.e., 3) from a starting number (e.g., 578) in a set period of time (22 seconds); (b) to report the result of each calculation aloud, so that they could be more focused on the calculation process which was occurring on-line and progressively; (c) to experience ascending and descending motions with the whole body, either in a passive (going up and down taking an elevator) or in an active (walking up and down the stairs) mode.

To summarize, we predict a congruency effect between the direction of the experienced motion and the spatial orientation inferred by the type of calculation made. More specifically, we hypothesize a facilitation for the congruent conditions (i.e., ascending body motion/upward orientation: additions; descending body motion/downward orientation: subtractions) with respect to the incongruent ones (i.e., descending body motion/upward orientation: additions; ascending body motion/downward orientation: subtractions). The specific hypothesis on congruent/incongruent conditions gathers from the concept of *groundedness* of numbers concepts proposed by Fischer [27]: “there is a universal association of small magnitudes with lower space and larger magnitudes with upper space” (*ibidem*, p. 162). Furthermore, Lakoff and Núñez [28] postulated that additions and subtractions can be conceptualized through the “*Arithmetic is Object Collection*” metaphor, according to which adding and subtracting numbers are understood as putting or taking away objects from collections. Thus, we expect a facilitation for additions, that imply an increase of quantity and therefore lead to larger numbers, with ascending motions and a facilitation for subtractions, that imply a decrease of quantity and therefore yield smaller numbers, with descending motions.

We also hypothesize a larger congruency effect when the calculations are performed simultaneously with the motion (on-line condition) with respect to when they have to be made after experiencing the motion (off-line condition).

Finally, we also test whether the congruency effect is influenced by the mode through which the motions are performed (i.e., passive, taking the elevator, or active, taking the stairs). In two recent researches, Hartmann and colleagues [29] [30] investigated whether the numerical processing was only influenced by active body movements or also by motions experienced in a passive fashion. In their studies, participants were seated in a chair positioned on a motion platform and were asked to generate numbers at random while the platform was moving along the transversal, frontal, and sagittal body planes (Experiment 1). Results indicated that the sensory self-motion cues, that is the information elicited by the passive body motions, were sufficient to interact with numerical cognition. More specifically, they found a bias for small numbers, that is small numbers were generated during leftward and downward motions as compared to rightward and upward motions, respectively.

Hence, the fact that in our study participants experience the motions through two different modes (i.e., passive and active) is a novelty with respect to the current literature. When asked to experience ascending/descending motions by taking the elevator (i.e., through a passive mode), participants experience a self perception of the direction of the motion (as in [29] [30]). Conversely, when the ascending/descending motion is experienced taking the stairs (i.e., through an active mode), participants perform an overt and real motor action with a full physical body involvement. Furthermore, it has to be pointed out that the sense of the motion direction differed in these two conditions. We typically experience the motion as fast and clearly vertical when using the elevator, while we perceive it as more progressive (i.e., the awareness of going up or down changes step by step) and less vertical for the stairs. Hence, we hypothesize that these two modes can have different impacts on the results. More specifically, we investigate whether a passive displacement of the body, but faster and vertical, is sufficient to obtain the congruency effect or whether the progressive and less vertical active body motion, which characterizes the stairs, is more efficient to obtain the congruency effect. In other words, if the congruency effect requires an active motor process, we should find the effect only in the stairs mode. Conversely, if the sense of a fast and vertical motion has a deeper impact on counting behavior, we can expect a congruency effect even when participants take the elevator, that is when the motions are experienced in a passive mode.

In case our predictions will be confirmed, interesting implications for the embodied and grounded cognition view can be drawn. According to this view, which emphasizes the continuity and the exchange between perception and action (e.g., [31] [32] [33] [34]), cognition is influenced by our previous experiences and it is constrained to specific physical characteristics of our body and of our sensory-motor system. Embodied cognition theories claim that both abstract and concrete concepts are grounded into perception-action systems. However, few evidence so far has shown how abstract concepts can be based on sensory-motor experiences (e.g., [35]; for a review, see [36]). Since one interesting example of abstract concepts is represented by numbers, studies on numerical cognition are highly relevant for the debate on embodied and grounded views (e.g., [14] [27]).

## Experiment 1

### Materials and Methods

#### Participants

Fifty-six students of the University of Bologna (30 females, mean age: 22 years) took part in the experiment and received 5 euro for their participation. The majority of participants had a background in humanities and they were all naïve as to the purpose of the experiment. Eight participants were eliminated and replaced from the same pool since they made more than 4 calculation errors (corresponding to the participants' errors mean plus one standard deviation).

#### Ethics Statement

The experiment was approved by the Psychology Department's ethical committee of the University of Bologna, and participants provided a written informed consent.

#### Apparatus and stimuli

Participants were asked to keep adding or subtracting 3 to a starting number (e.g., 371) for 22 seconds and to say the result of each calculation aloud (e.g., 374, 377, 380 or 368, 365, 362 and so on, for additions and subtractions, respectively, until the 22 seconds were elapsed). We made sure that the starting numbers: a) were always composed by three digits (e.g., 371; 587); b) started with two different digits (i.e., 3 or 5, such as 371 or 588).

### Procedure

Participants were required to make the calculations (additions or subtractions) while (on-line condition) or after (off-line condition) taking the elevator (passive mode) or taking the stairs (active mode). In other words, half of the participants were asked to make the calculations while taking the elevator and the stairs (on-line condition), whereas the other half had to make additions and subtractions after the elevator or the stairs were taken (off-line condition).

In order to keep the active and passive motion modes separated, each condition included two blocks (whose order was counterbalanced between subjects): in one block participants performed the calculations while taking the elevator or just after it had been taken, whereas in the other block calculations were performed while taking the stairs or just after they had been taken. Within each block, participants were required to perform four trials, resulting from the combination of the two types of calculation (i.e., additions and subtractions) and the two types of motion (i.e., ascending and descending). We designed each block in order to make additions and subtractions always alternate (i.e., an addition always followed a subtraction and vice versa).

At the beginning (for on-line condition) or at the end (for off-line condition) of the motion, the experimenter spoke the starting number aloud and a go signal followed. Immediately after the go signal, the participant had to repeat the starting number and then to keep speaking aloud the result of each calculation for 22 seconds consecutively until a stop signal was given. Therefore, the number of calculations made within the 22 seconds window entirely depended on the participants' calculation speed. If participant made a calculation error, the trial was stopped and a new trial started over choosing a different starting number. No feedback of any kind was given during the calculations. Instructions stressed the importance of accuracy over speed.

The experimenter was always present during the whole experiment. For the passive mode, the experimenter went up/down using the elevator together with the participant. For the active mode, she walked close to the participant while going up/down the stairs and asked the participant to keep her pace throughout the whole movement. In other words, the participant and the experimenter went up/down the stairs together.

Responses were recorded by the experimenter who kept track and note of the starting number assigned to the participants and of the final number reached at the end of the 22 sec time window.

Participants were thanked and debriefed at the end of the experiment.

### Results and Discussion

The number of calculations made within the 22 seconds time window was used as our dependent variable. We predicted a congruency effect between the direction of the experienced motion and the type of calculation made (implying an upward or downward orientation for additions and subtractions, respectively). For this reason, we divided the trials in congruent (ascending motions–additions; descending motions–subtractions) and incongruent (ascending motions–subtractions; descending motions–additions), and then we averaged the number of calculations separately for each group of pairings. A repeated-measures ANOVA on correct calculations was thus conducted with *Condition* (on-line vs. off-line) as between-subjects factor, and *Congruency* (congruent vs. incongruent) and *Mode* (elevator-passive motion mode vs. stairs-active motion mode) as within-subjects factors.

The *Congruency* [*F*(1,54)  = 6.16, *MSE*  = 0.84, *n^2^_p_*  = 0.10, *p<*.05] and *Mode* [*F*(1,54)  = 29.63, *MSE*  = 3, *η_p_* ^2^  = 0.35, *p<*.001] factors were significant, while the *Condition* factor was not [*F<*1]. The number of calculations was higher when participants performed: a) congruent pairings (M = 11) with respect to incongruent ones (M = 10.7); b) the task in a passive (M = 11.5) with respect to an active (M = 10.3) mode. The *Condition* x *Mode* interaction was significant [*F*(1,54)  = 14.61, *MSE* = 3, *n^2^_p_* = 0.21, *p<*.001]. Fisher's LSD post-hoc test showed that, in the on-line condition, the number of calculations was higher for the passive than for the active mode (Ms = 12 and 9.8, respectively, *p<*.001), while in the off-line condition no difference emerged between passive and active mode (Ms = 11 and 10.7, respectively, *p = *.3), see Table 1.

**Table 1:** Number of calculations of Experiment 1 as a function of *Condition* (on-line vs. off-line) and *Congruency* (congruent vs. incongruent) for the passive (a) and active (b) mode.

	Condition	Congruency	Number of calculations	
**(a) Passive mode (elevator)**	on-line	congruent	12.5	Additions 13.07
				Subtractions 11.93
	on-line	incongruent	11.5	Additions 12.43
				Subtractions 10.54
	off-line	congruent	11.1	Additions 11.71
				Subtractions 10.43
	off-line	incongruent	11	Additions 11.82
				Subtractions 10.21
**(b) Active mode (stairs)**	on-line	congruent	9.9	Additions 10.46
				Subtractions 9.25
	on-line	incongruent	9.8	Additions 10.36
				Subtractions 9.32
	off-line	congruent	10.7	Additions 11.5
				Subtractions 9.97
	off-line	incongruent	10.6	Additions 11.4
				Subtractions 9.82

The last column indicated the number of calculations keeping separate the addition and subtraction.

The three-way *Congruency* x *Mode* x *Condition* interaction was not significant, [*F*(1,54)  = 3.17, *MSE* = 1.27, *n^2^_p_* = 0.05, *p* = .08].

**Figure 1:**
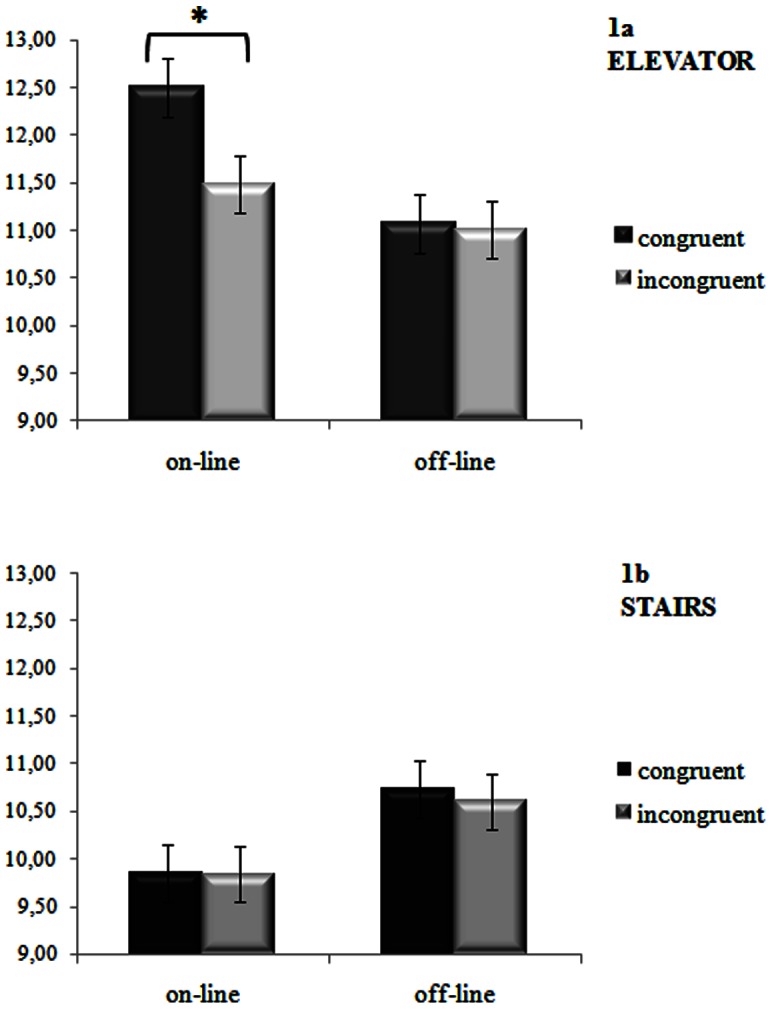
Number of calculations for congruent (ascending motion–additions; descending motion–subtractions) and incongruent pairings (ascending motion–subtractions; descending motion–additions) performed during (on-line condition) or after (off-line condition) experiencing the motions through a passive (i.e., elevator, panel 1a) or active (i.e., stairs, panel 1b) mode. Bars are standard error of the mean.

In order to better investigate the significant *Condition x Mode* interaction, separate ANOVAs by levels of *Mode* were run. When the motion was experienced through the passive mode, that is taking the elevator, the *Congruency* factor was significant [*F*(1,54)  = 6.73, *MSE*  = 1.19, *n^2^_p_* = 0.11, *p<*.05], while the *Condition* factor was not (*p* = .26). The number of calculations was higher when participants performed congruent (M = 11.8) with respect to incongruent pairings (M = 11.3), see Table 1. Crucially, the *Congruency* x *Condition* interaction was significant [*F*(1,54)  = 5.45, *MSE*  = 1.19, *n^2^_p_* = 0.09, *p<*.05]. Fisher's LSD post-hoc test showed that, in the on-line condition, the number of calculations was higher for congruent pairings than for incongruent ones (Ms = 12.5 and 11.5, respectively, *p*<.001, see Figure 1 panel a, and Table 1).

Conversely, when the movement was experienced through an active mode no significant main effects and interaction were found (*F_s_ <1*), (see Figure 1 panel b, and Table 1).

In line with our hypothesis, results demonstrated a congruency effect between the direction of the experienced motion and the orientation inferred by the type of calculation made. Indeed, a facilitation was found for the congruent conditions (i.e., ascending motion/upward orientation: additions; descending motion/downward orientation: subtractions) over the incongruent conditions (i.e., ascending motion/downward orientation: subtractions; descending motion/upward orientation: additions) when the motions were simultaneous with the calculation process (i.e., on-line condition) and were experienced trough a passive mode (i.e., taking the elevator). Furthermore, the lack of significant results for the off-line condition suggests that the simultaneity between the experienced motions and the calculation processes is a crucial factor for the congruency effect to emerge, that we attribute to the emergence of a proper embodied simulation.

The lack of a congruency effect for the active mode could be due to different factors. First of all, as anticipated above, in the stairs mode the sense of the motion was more progressive and less vertical with respect to the elevator mode in which the motion was perceived as faster and more vertical. Another explanation could be participants' performance was influenced by a dual task. In other words, the motor actions of going up or down the stairs may have interfered with the simultaneous calculations processes. This explanation may also account for the result of the *Mode* factor, which indicates that fewer additions and subtractions were made when participants walked up/down the stairs with respect to when the elevator was taken. However, it is worth noting that a dual task could have caused just a general decrease in performance (as shown by *Mode* factor) without selectively changing the congruency advantage.

One could argue that the congruency effect yielded in the passive mode can be due either to spatial and perceptual features (i.e., the feeling of going up or down) or to a more abstract representation of the motion direction, rather than to motoric features. In order to test these alternative explanations, we carried out a further experiment in which participants were required to make additions and subtractions while just imagining to take the elevator or the stairs. Specifically, participants had to perform calculations simultaneously to the imagined motion and, thus, they were not required to perform a motor task. In other words, we got rid of the body effort during the calculation processes, hence eliminating the possible dual task due to motor actions. Our predictions are as follows: if the congruency effect reported for the elevator mode in the previous experiment was spatial or perceptual, then a similar effect should also be yielded in this new experiment. Conversely, no effect should be found if the congruency effect was due to motoric factors.

In light of the significant result registered in the on-line condition of Experiment 1, in the following experiment we will not take more into account the differences between to make additions or subtractions while performing (on-line condition) or after having experienced (off-line condition) a motion, but we will focus on an imaging on-line condition.

## Experiment 2

### Materials and Methods

#### Participants

Thirty new students (16 females, mean age: 20.4 years) from the same pool were selected. Two participants were eliminated because they made more than 4 calculation errors (corresponding to the participants' errors mean plus one standard deviation).

#### Ethics Statement

The experiment was approved by the Psychology Department's ethical committee of the University of Bologna, and participants provided a written informed consent.

#### Apparatus, stimuli and procedure

Apparatus, stimuli and procedure were the same as in Experiment 1. The only difference was that participants were asked to make the calculations (additions or subtractions) while imagining to perform an upward or downward motion.

As for Experiment 1, participants performed 2 blocks of 4 trials each. Each block corresponded to a specific mode (i.e., participants were to ask to imagine to take the elevator in one block and to take the stairs in the other, always while counting.). Before each block, the experimenter and the participant experienced an upward and downward motion taking the elevator or the stairs (depending on the first block to be performed). This was done to allow them to experience the same motions performed by the participants of Experiment 1. After experiencing the motions, the experimenter took the participant in a different room in which the experiment was performed. At the beginning of each trial, the experimenter asked the participant to close her eyes and imagine being in the elevator/on the stairs, at the lowest/highest floor, and more specifically, the same elevator and stairs they had just taken. She explained that, immediately after the go signal, the participant had to start to imagine to going up/down in the elevator/stairs, and at the same time to repeat the starting number and then to keep imagining and speaking aloud the result of each calculation for 22 seconds consecutively, until a stop signal was given. When the first 4 trials were over, the experimenter and the participant experienced the upward and downward motions using the other mode (the elevator or the stairs), and then performed the remaining 4 trials following the same procedure.

### Results and Discussion

A repeated-measures ANOVA on correct calculations was conducted with *Congruency* (congruent vs. incongruent) and *Mode* (elevator-passive motion mode vs. stairs-active motion mode) as within-subjects factors.

Neither the *Congruency* and *Mode* factors [*Fs* <1], nor their interaction [*F*(1,27)  = 2.12, *MSE*  = .51, *η_p_* ^2^  = 0.07, *p = *.16] were significant, indicating that the congruency effect was not yielded when the ascending/descending motions were just imagined rather than actually performed.

In order to compare the outcomes of Experiment 1 with those of Experiment 2, we ran a further ANOVA with *Task* (Experiment 1-motion real vs. Experiment 2-motion imagined) as between-subjects factor, and *Congruency* (congruent vs. incongruent) and *Mode* (elevator-passive motion mode vs. stairs-active motion mode) as within-subjects factors. In order to investigate the conditions in which the motions and the calculations were performed simultaneously, only data of the on-line condition of Experiment 1 were considered.

The *Task* factor was not significant [*F*(1,54)  = 1.28, *MSE*  = 37.61, *η_p_* ^2^  = 0.02, *p = *.26]. The *Congruency* [*F*(1,54)  = 6.15, *MSE*  = .74, *η_p_* ^2^  = 0.10, *p = *.02], and the *Mode* [*F*(1,54)  = 21.59, *MSE*  = 3.23, *η_p_* ^2^  = 0.29, *p<*.001] factors were significant. The first result showed that participants made more calculation in the congruent (M = 10.6) than in the incongruent (M = 10.3) condition. The latter indicated that the number of calculations was higher when participants performed the task in a passive (M = 11) with respect to an active (M = 9.9) mode, see Table 2. The *Task* x *Congruency* [*F*(1,54)  = 4.06, *MSE*  = 0.74, *n^2^_p_*  = 0.07, *p*<.05], and the *Task* x *Mode* [*F*(1,54)  = 18.28, *MSE*  = 3.23, *n^2^_p_*  = 0.25, *p<*.001] interactions were significant. Fisher's LSD post-hoc test showed that, when the motion was actually experienced (Experiment 1), the number of calculations was higher for (a) the congruent than for the incongruent condition (Ms = 11 and 10.7, respectively, *p*<.001) (b) the passive mode with respect the active mode (Ms  = 12 and 9.8, respectively, *p*<.001). Conversely, when the motion was just imagined (Experiment 2), no differences emerged between the congruent and incongruent conditions (Ms  = 10 and 10, respectively, *p* = .74) and between the passive and the active mode (Ms  = 10 and 9.9, respectively, *p* = .79). Moreover, the passive mode of Experiment 1 yielded a higher number of calculation with respect of the passive mode of Experiment 2 (Ms  = 12 and 10 respectively, *p*<.05). The *Mode* x *Congruency* [*F*(1,54)  = 1.38, *MSE*  = 0.93, *n^2^_p_*  = 0.02, *p* = .24] interaction was not significant.

**Table 2:** Number of calculations of the Experiment 2 as a function of *Congruency* (congruent vs. incongruent) for the passive (**a**) and active (**b**) mode.

	Congruency	Number of calculations	
**(a) Passive mode (elevator)**	Congruent	10	Additions 10.64
			Subtractions 9.29
	incongruent	10.1	Additions 10.64
			Subtractions 9.57
**(b) Active Mode (stairs)**	congruent	10.1	Additions 10.79
			Subtractions 9.36
	incongruent	9.8	Additions 10.54
			Subtractions 9.11

The last column indicated the number of calculations keeping separate the addition and subtraction.

**Figure 2:**
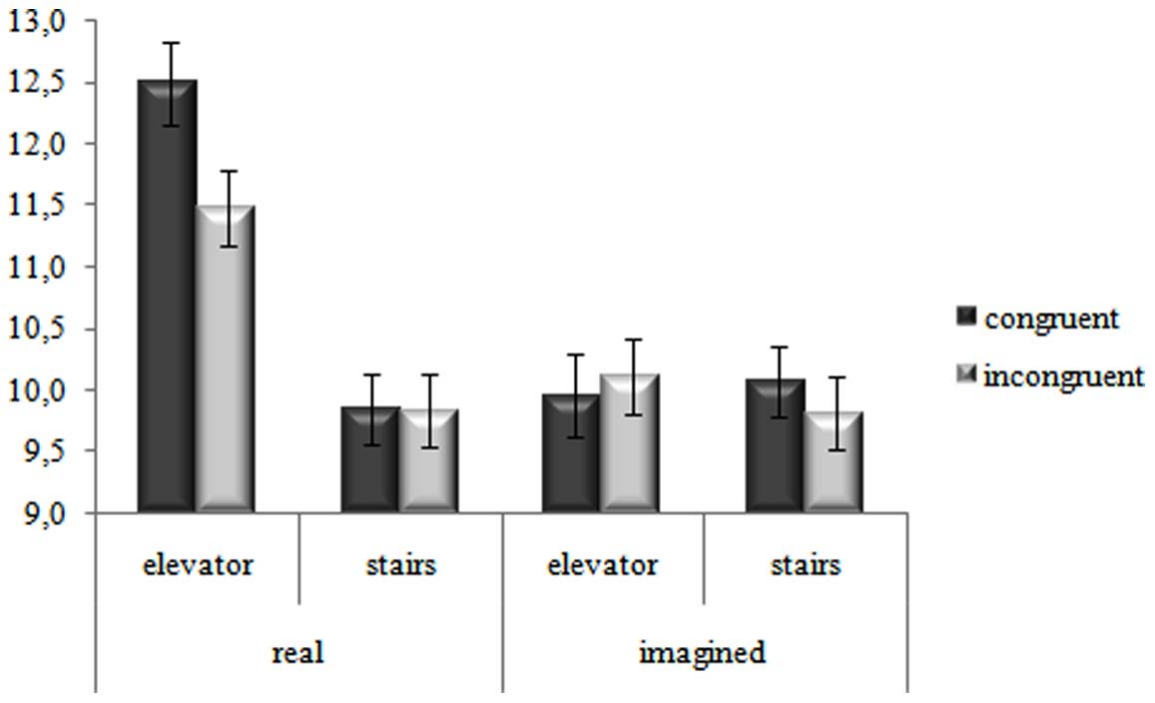
Number of calculations for congruent (ascending motion–additions; descending motion–subtractions) and incongruent pairings (ascending motion–subtractions; descending motion–additions) performed experiencing (i.e., real) or imagining (i.e., imagined) the motions through a passive (i.e., elevator) or active (i.e., stairs) mode. Bars are standard error of the mean.

Interestingly, the three-way interaction was significant [*F*(1,54)  = 7.27, *MSE*  = 0.93, *n^2^_p_*  = 0.12, *p*<.01]. Fisher's LSD post-hoc test confirmed the pattern of results found for the on-line condition of Experiment 1, see Figure 2. Furthermore, participants performed the highest number of calculations with congruent pairings in the passive mode of Experiment 1(M = 12.5, *p_s_* <.05). Crucially, in Experiment 2, that is when the movement was just imagined, no difference emerged between congruent and incongruent pairings, either in the passive (Ms  = 10 and 10.1, respectively, *p = *.58), and in the active mode (Ms  = 10.1 and 9.8, respectively, *p = *.34), see Figure 2.

Experiment 2 was run in order to clarify if the lack of the congruency effect for the stairs mode found in Experiment 1 could be due to a dual task (going up and down the stairs while counting). Results showed that, first, the congruency effect failed to emerge overall when the task of going up/down the stairs was just imagined and not actually experienced. Second, no differences were found in terms of congruency and number of calculations among the stairs and the elevator mode, differently from Experiment 1.

Two main conclusions can be drawn from these results. First, the motion of the whole body need to be actually experienced and not only imagined for the congruency effect to emerge. That is, we found a significant effect only when participants actually experienced ascending/descending motions and not when they just imagined the motions. Hence, we can claim that the congruency effect obtained in Experiment 1 was motoric rather than spatial and/or perceptual. This finding is in line with the studies which demonstrate a motor-to-semantic effect, that is a consistent influence of the motor processes over the semantic ones [21] [22] [23] [37].

Second, the congruency effect emerged only when the direction of the motion was experienced through the elevator mode, having a clearly vertical and fast perception of the motion. Although this motion experience did not involve any intention to move nor any overt motor activity, it is sufficient to interact with calculation processes and thus, more in general, to influence numerical cognition.

## General Discussion

The present study investigates how experiencing a self perception of the direction of the motion (i.e., passive mode) and experiencing an overt and real motor action (i.e., active mode) can influence the orientation inferred by the type of calculations made.

Two main conclusions can be drawn from our findings. First, we found a congruency effect between the body motions and the calculation processes, instead of a given set of numbers. Hartmann et al. ([29] Experiment 1; see also [30]) demonstrated that the experience of horizontal and vertical motions with the whole body (i.e., participants were moved in different directions while seated in a chair) influenced numerical cognition, shifting attention along the MNL and also modulating the magnitude of self-generated numbers. Interestingly, our results indicate that ascending/descending passive body motions influenced not only the number generation process, but also the processes that can lead participants to represent numbers as magnitude with an upward and downward orientation, that is the arithmetical calculations of addition and subtraction. This suggests that not only the absolute numerical information (e.g., 2 rather than 9) and its numerical magnitude, but also the processes leading to the numerical magnitude are strongly connected with the processing of spatial information.

The second conclusion regards the debated issue of the processes underlying the spatial representation of numbers [14]. The congruency effect we found in our experiment may be based indeed on an attention shifting mechanism (i.e., the numbers representation directs visuospatial attention to a congruent spatial location, see [12] [38]) or, alternatively or in addiction, on a simulation process (i.e., a process that entails the recruitment of the same neurons that are activated during the real first-person experience of the situation, action, emotion, object or entity mentioned; see [39] [40] [2] [41] [42]). We argue that the second option could explain our results better, even if we acknowledge that there is a large amount of evidence in favor of the attention shifting account (for a review, see [1]). Indeed, if the spatial representation of numbers magnitude was entirely due to the spatial orientation of attention, then we should have found the congruency effect also when additions and subtractions were calculated after experiencing the real motion (off-line condition of Experiment 1) and when those calculations were performed while participants just imagined the motions (Experiment 2). But these were not the cases, as we found the effect only when real body motions were experienced and the calculations were performed while moving.

## Conclusions

Aim of the present study was to tackle the issue of whether calculation processes, such as additions and subtractions, are influenced by real motions experienced with the whole body. Results spoke in favor of a close connection between numbers, space and motor processes, indicating that numbers are represented as magnitudes implying an upward and downward orientation, in line with an embodied and grounded perspective. Importantly, our findings are in line with Fischer and Brugger's [2] proposal which explains the origin of the Spatial-Numerical Associations (SNAs) acknowledging the grounded and embodied nature of numerical cognition, which would emerge in fact from finger counting. The current study provides further evidence in favor of an embodied nature of number processing, showing that sensory-motor interaction, led by the whole body motion, can influence numbers representation.

In conclusion, our results have broad implications for different lines of research, suggesting that our everyday activities, as movements in real-life situations, are likely to interact with higher-order cognitive processes, as spatial representation and number processing. We move our body daily and, thanks to movements, we develop as autonomous entities able to explore the environment. Our study contributes in showing that the basic ability to move our body, which we share with other animals, grounds at the basis of sophisticated and probably exquisitely human abilities, such as that of counting.
